# Alteration of tumor characteristics following neoadjuvant treatment in gastric adenocarcinoma: a case report

**DOI:** 10.3389/fonc.2026.1692675

**Published:** 2026-01-29

**Authors:** Lingzhi Peng, Zhanying Ma, Yunfei Zhao, Hong Ma, Weijun Liu, Ming Huang

**Affiliations:** 1 Department of Surgical Oncology, Gansu Provincial Hospital, Lanzhou, China; 2 The First School of Clinical Medicine, Gansu University of Traditional Chinese Medicine, Lanzhou, China; 3 Department of Interventional Oncology, Gansu Provincial Hospital, Lanzhou, China; 4 Department of General Surgery, Longxi County First People’s Hospital, Longxi, China; 5 Department of Neurosurgery, Gansu Provincial Hospital, Lanzhou, China

**Keywords:** chemotherapy, gastric cancer, gastric neuroendocrine tumor, neoadjuvant therapy, tumor biological behavior

## Abstract

A 69-year-old female was admitted to the hospital with abdominal pain and distension. Gastroscopy revealed an irregular protruding lesion on the lesser curvature of the gastric fundus, and pathological examination indicated moderately to poorly differentiated adenocarcinoma. Enhanced abdominal computed tomography (CT) identified a space-occupying lesion at the gastric fundus with multiple enlarged lymph nodes. The initial diagnosis was moderately to poorly differentiated adenocarcinoma, cT3N3M0 (Stage IIIa). The patient subsequently underwent neoadjuvant therapy comprising albumin-bound paclitaxel, camrelizumab, oxaliplatin, and S-1.After four cycles, the tumor significantly reduced in size, with restaging at yT2N2M0 (Stage IIb). Following multidisciplinary team (MDT) consultation, the patient underwent radical total gastrectomy with esophagojejunostomy. Postoperative recovery was uneventful, and the patient was discharged in improved condition. Notably, postoperative pathological analysis revealed a neuroendocrine tumor, demonstrating an alteration in tumor histology after neoadjuvant therapy for gastric cancer (GC).

## Introduction

Since Wilke et al. first reported the efficacy of neoadjuvant therapy in gastric cancer (GC) in 1989 (1), it has become a critical preoperative approach for patients with locally advanced GC, enhancing conditions for surgical resection. As a significant therapeutic strategy in oncology, neoadjuvant therapy aims to reduce tumor size, achieve downstaging, increase surgical success rates, and improve patient survival (2). Currently, novel therapeutic approaches, such as chemotherapy, immunotherapy, targeted therapy, and combined modalities, have expanded therapeutic options but also raised new clinical challenges. Here, we report a rare case in which the tumor histological nature changed following neoadjuvant therapy.

## Case presentation

Chief Complaint: Abdominal pain and distension for over 3 months, with symptom exacerbation over the past week.

Present History: On July 5, 2023, the patient experienced intermittent abdominal pain and distension without any apparent cause, predominantly located in the upper abdomen, accompanied by fatigue and poor appetite, without nausea, vomiting, acid reflux, or heartburn. No specific treatment was administered at that time. One week prior to admission, symptoms worsened, prompting the patient to visit Longnan Hospital of Traditional Chinese Medicine, where gastroscopy revealed an elevated lesion at the lesser curvature of the gastric fundus. Biopsy indicated moderately to poorly differentiated adenocarcinoma. Seeking further treatment, the patient was admitted to the Department of Surgical Oncology, Gansu Provincial People’s Hospital, on July 15, 2025, with a diagnosis of malignant tumor of the gastric fundus. During the illness course, the patient maintained clear consciousness, good mental status, normal dietary intake, and regular bowel and urinary functions. The patient reported a weight loss of approximately 5 kg since symptom onset.

Past Medical History: On November 15, 2018, the patient presented with paroxysmal colicky pain in the right upper abdomen without any obvious cause. The pain radiated to the right shoulder and back, accompanied by nausea and vomiting. The patient reported no fever, chills, jaundice of the skin or sclera, abdominal distension, or diarrhea. Following admission to the Department of Oncologic Surgery at Gansu Provincial People’s Hospital, relevant laboratory tests and imaging examinations were completed, leading to a diagnosis of gallstones complicated by acute cholecystitis. After ruling out surgical contraindications, laparoscopic cholecystectomy was performed. Postoperative treatment included anti-infection therapy, fluid replacement, and nutritional support. The patient’s condition improved, and the patient was discharged in stable condition. There was no history of other surgeries or significant medical conditions.

Physical Examination: The abdomen was flat, with mild tenderness in the upper abdomen. There was no rebound tenderness or muscular rigidity, and other physical examination findings were unremarkable.

Family History: No family history of malignancy or genetic diseases reported.

**Figure 1:**
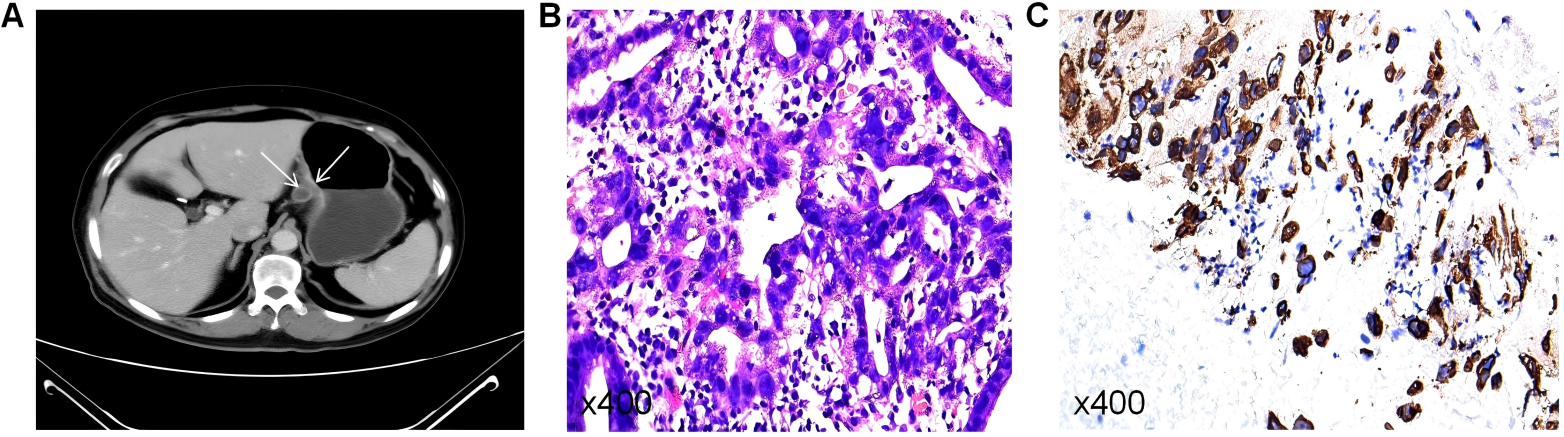
**(A)** Enhanced CT before neoadjuvant therapy. **(B)** Preoperative HE staining indicating moderately to poorly differentiated adenocarcinoma. **(C)** Positive CK8/18 staining (+).

Imaging Examination: Abdominal enhanced CT revealed localized gastric wall thickening along the lesser curvature of the gastric fundus, involving approximately 7 cm of gastric wall. The serosal surface at the lesion site appeared rough, with significant and homogeneous enhancement on the contrast-enhanced scan. Multiple mildly enhanced lymph nodes were observed in the hepatogastric space, with the largest measuring 1.4 cm in short diameter. The imaging diagnosis indicated a space-occupying lesion on the lesser curvature of the gastric fundus with multiple enlarged lymph nodes (**Figure 1A**). Routine gastroscopy demonstrated a protruding lesion at the same site, and biopsy pathology confirmed moderately to poorly differentiated adenocarcinoma (**Figure 1B**) with positive staining for CK8/18 (**Figure 1C**).

Final Diagnosis: Moderately to poorly differentiated adenocarcinoma of the gastric fundus (lesser curvature side), cT3N2M0 (Stage IIIa).

BMI: 22 kg/m2; Body surface area: 1.51 m2; ECOG: 0.

Imaging examinations: Abdominal enhanced CT revealed localized thickening of the gastric wall at the lesser curvature of the gastric fundus, involving approximately 7 cm of the gastric wall, with a rough serosal surface. The lesion showed significant and homogeneous enhancement. Multiple mildly enhanced lymph nodes were observed in the hepatogastric space, with the largest node measuring 1.4 cm in short diameter. CT findings suggested an occupying lesion at the lesser curvature of the gastric fundus with multiple enlarged lymph nodes (**Figure 1A**). Routine gastroscopy identified an elevated lesion at the lesser curvature of the gastric fundus. Pathological examination confirmed moderately to poorly differentiated adenocarcinoma (**Figure 1B**), with positive CK8/18 staining (**Figure 1C**).

Final diagnosis: Moderately to poorly differentiated adenocarcinoma at the lesser curvature of the gastric fundus, clinical stage cT3N2M0 (stage IIIa).

Abnormal tumor marker results prior to neoadjuvant therapy (**Table 1**).

**Table 1:** Abnormal tumor marker results prior to neoadjuvant therapy.

Cancer biomarkers	Result	Trend	Reference range	Unit
AFP	32.36	↑	<8.78	ng/mL
CEA	10.34	↑	<5	ng/mL
CA242	29.04	↑	<25	U/ml

BMI: 22 kg/m2, Body surface area: 1.51 m2, ECOG: 0.

After multidisciplinary team (MDT) discussions, the patient received four cycles of neoadjuvant therapy. During treatment, the patient demonstrated good tolerance to chemotherapy, experienced no significant discomfort or severe adverse effects, maintained high adherence, and completed each treatment cycle as scheduled (**Table 2**).

**Table 2:** Preoperative medication schedule.

Date\Medication	Oxaliplatin	S-1(Tegafur, Gimeracil, and Oteracil Potassium Capsules)	Camrelizumab	Albumin-bound paclitaxel
First(2023.07. 18)	150mg.iv.gtt.d1	50.po.bid.d1-d14	200mg.iv.gtt.d1	250mg.iv.gtt.d1
Second(2023.08.08)	150mgiv.gtt.d1	50.po.bid.d1-d14	200mg.iv.gtt.d1	250mg.iv.gtt.d1
Third(2023.08.29)	150mgiv.gtt.d1	50.po.bid.d1-d14	200mg.iv.gtt.d1	250mg.iv.gtt.d1
Fourth(2023.09.21)	150mg.iv.gtt.d1	50.po.bid.d1-d14	200mg.iv.gtt.d1	250mg.iv.gtt.d1
Fifth(2023.10.29)	Radical total gastrectomy with esophagojejunostomy and peritoneal lymphadenectomy			

Outcome and Follow-up: After completion of the fourth cycle (preoperative), tumor markers were reassessed; abnormal results are summarized in **Table 3**.

**Table 3:** Abnormal tumor marker results after neoadjuvant therapy.

Cancer biomarkers	Results	Trend	Reference range	Unit
CEA	11.4	↑	<5	ng/mL
PGI	40.3	↓	>45	ng/mL
CA72-4	10.3	↑	<1	U/ml

**Figure 2:**
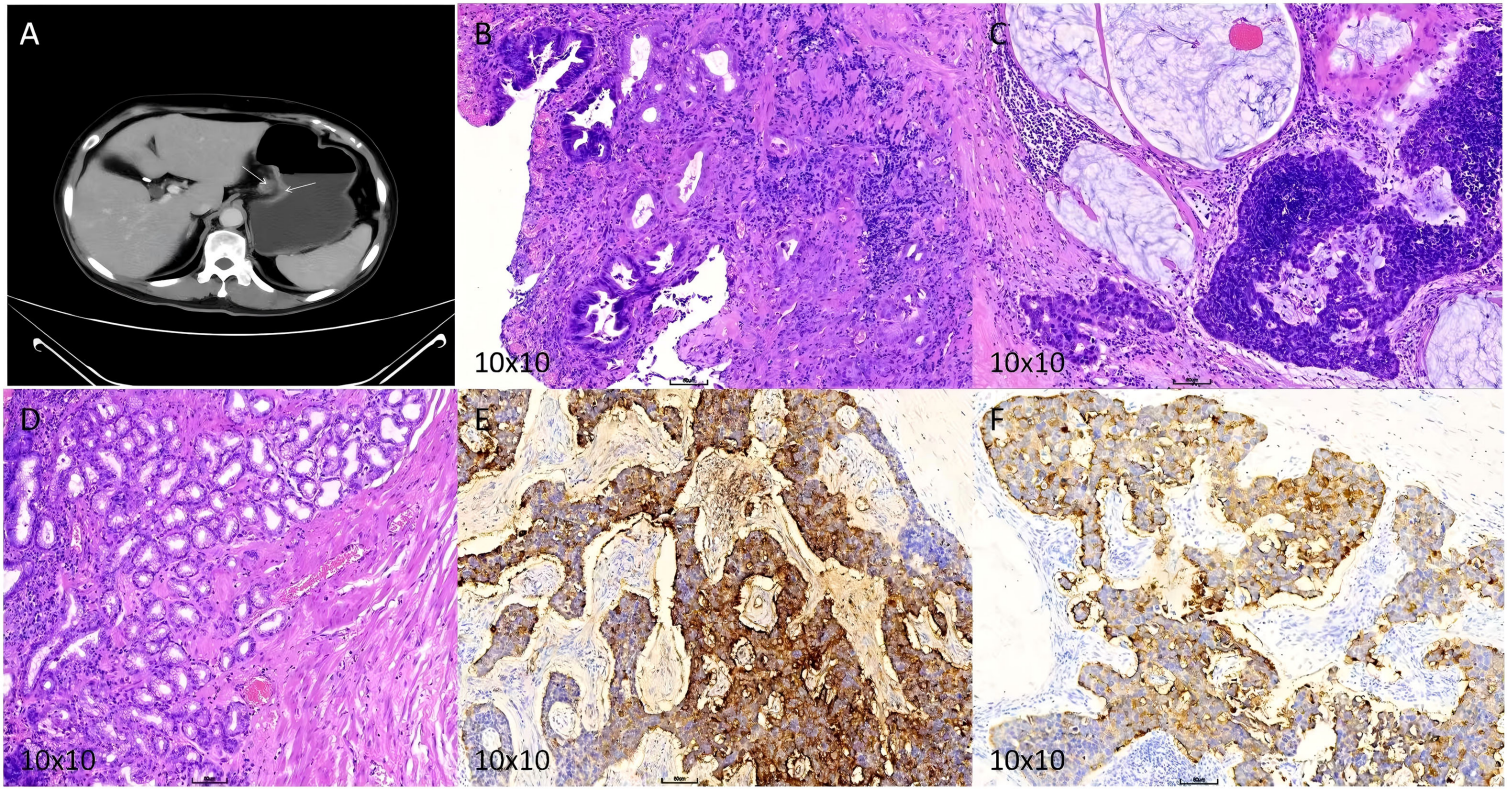
**(A)** Enhanced CT after neoadjuvant therapy. **(B)** Postoperative HE staining of primary lesion. **(C)** Postoperative HE staining of lymph nodes. **(D)** HE staining of adjacent normal tissues. **(E)** Positive CgA staining in lymph nodes. **(F)** Positive Syn staining in lymph nodes.

Enhanced abdominal CT indicated a reduction in tumor size (**Figure 2A**), with a revised staging of yT2N2M0 (Stage IIB). Following further MDT consultation, the patient underwent radical total gastrectomy with Roux-en-Y esophagojejunostomy. Intraoperative exploration revealed no peritoneal ascites, and no nodules were detected in the liver, spleen, kidneys, greater omentum, abdominal wall, or pelvic cavity. The gallbladder was absent, and notable adhesions and edema were present between the greater omentum and pelvic abdominal wall. Prominent enlarged lymph nodes were identified along the lesser curvature side of the gastric fundus. Postoperative pathological findings (**Figure 2B**) indicated focal ulcer formation in the gastric mucosa, surrounded by moderate chronic inflammation with moderate activity, moderate atrophy, and mild intestinal metaplasia. Some glands exhibited low-grade intraepithelial neoplasia, but no definitive adenocarcinoma components were observed. Additionally, tumor metastasis characterized as neuroendocrine tumor was identified in several lymph nodes within the subserosal fibrous tissue (**Figure 2C**). Immunohistochemical staining of adjacent normal tissues and involved lymph nodes revealed positive expression of CgA (**Figure 2E**) and Syn (**Figure 2F**). Postoperatively, the patient underwent four cycles of chemotherapy with etoposide and cisplatin (**Table 4**). After the first postoperative chemotherapy cycle, the patient developed grade IV myelosuppression (WBC: 0.87×109/L, NE: 0.03×109/L, PLT: 26×109/L) and was treated with red blood cell and platelet transfusions, resulting in clinical improvement. Subsequent chemotherapy cycles were administered at reduced dosages, with the patient exhibiting good tolerance and completing all planned treatments successfully. Since the final postoperative treatment on February 18, 2024, the patient has been regularly followed up for over 18 months. Abdominal enhanced CT scans showed no abnormalities, recurrence, or metastasis.

**Table 4:** Postoperative medication schedule.

Date\Medication	Etoposide	Cisplatin
First(2023. 11.22)	150mg.iv.gtt.d1-d3	110mg.iv.gtt.d1
Second(2023. 12.22)	120mg.iv.gtt.d1-d3	90mg.iv.gtt.d1
Third(2024.01.09)	120mg.iv.gtt.d1-d3	90mg.iv.gtt.d1
Fourth(2024.02. 18)	120mg.iv.gtt.d1-d3	90mg.iv.gtt.d1

## Discussion

According to the latest global cancer statistics, GC remains among the most prevalent malignancies of the digestive system, ranking fifth in global cancer incidence and mortality (3). Although the incidence and mortality rates of GC have shown declining trends in recent years, GC continues to pose a significant threat to human health and quality of life (4, 5). While the 5-year survival rate for early-stage GC can exceed 90%, over 80% of patients are diagnosed at advanced stages due to the nonspecific nature of clinical symptoms. Consequently, the 5-year survival rate for advanced GC remains approximately 31% (6, 7), indicating an unfavorable prognosis. With ongoing advancements in research, neoadjuvant therapy has expanded treatment options for patients with advanced GC, significantly improving surgical resection rates and long-term survival (6). For example, the randomized, open-label phase III RESOLVE trial demonstrated that combining neoadjuvant therapy with surgical intervention markedly prolonged patient survival, achieving a 5-year survival rate of approximately 60% (8). Despite these advantages, the broader adoption of neoadjuvant therapy has introduced new clinical challenges.

In the current case, the tumor exhibited altered histological characteristics following neoadjuvant therapy. Although the underlying mechanisms remain unclear, several hypotheses may explain this phenomenon: (1) Tumor heterogeneity: Gastric adenocarcinomas frequently contain subclones exhibiting distinct differentiation pathways, including occult neuroendocrine components (9–11). Neoadjuvant therapy may selectively eradicate treatment-sensitive adenocarcinoma cells, allowing resistant neuroendocrine cells to proliferate and become dominant under selective therapeutic pressure (12). (2) Preoperative misdiagnosis: In mixed tumor cases, adenocarcinoma or squamous cell carcinoma components are typically superficial and easily accessible by endoscopic biopsy. Conversely, neuroendocrine components often reside in deeper gastrointestinal layers and are thus less likely to be sampled, leading to preoperative diagnostic omissions (13–15). Therefore, only adenocarcinoma may be diagnosed initially, with neuroendocrine components overlooked. Deep biopsy should be considered when imaging examinations suggest the possibility of neuroendocrine differentiation. (3) Selective effects of chemotherapy: Chemotherapeutic agents, particularly platinum-based drugs and fluoropyrimidines, primarily target rapidly proliferating adenocarcinoma cells, potentially sparing slower-growing neuroendocrine populations (16–18). In contrast, neuroendocrine carcinoma cells typically exhibit slower proliferation rates and may progressively develop resistance to neoadjuvant chemotherapy, ultimately surviving and proliferating after treatment (12, 19, 20). (4) Driving effects of immunotherapy: Neoadjuvant immune checkpoint inhibitors activate T cells to eliminate tumor cells. However, in neuroendocrine tumors, high PD-L1 expression is significantly correlated with tumor grade and shorter survival, suggesting limited efficacy of immunotherapy in these tumors (21, 22). Immunotherapy may preferentially target highly immunogenic adenocarcinoma cells, indirectly facilitating the selective expansion of neuroendocrine cells. (5) Lineage reprogramming: Under therapeutic pressure, adenocarcinoma cells may undergo epithelial-neuroendocrine transdifferentiation. The NOTCH signaling pathway, essential for maintaining glandular epithelial differentiation, may become inactivated by neoadjuvant therapy (23). For instance, in prostate cancer, activation of this pathway can inhibit neuroendocrine differentiation in advanced disease and modify the tumor immune microenvironment, while its inhibition promotes neuroendocrine differentiation (24). To date, this mechanism has not been documented in gastric malignancies. Neoadjuvant therapy may induce mutations in the TP53 and RB1 genes. TP53 primarily initiates cellular repair or apoptosis upon DNA damage, whereas RB1 serves as a major regulator of the G1/S checkpoint, preventing abnormal cell proliferation. When mutations occur in TP53 and RB1, cells exhibit increased lineage plasticity, losing their original differentiation features and gaining the potential to transdifferentiate into other cell types. Additionally, treatment-related damage to the tumor microenvironment might accelerate the accumulation of these mutations. In studies investigating the conversion to neuroendocrine tumors following neadjuvant therapy for prostate and lung cancers, prostate cancer patients treated with docetaxel and androgen receptor antagonists may transform into neuroendocrine prostate cancer. Similarly, patients with EGFR-mutant lung adenocarcinoma may convert to small cell lung cancer after targeted treatment with EGFR-TKIs such as gefitinib, erlotinib, and osimertinib. During malignant tumor treatment, if rapid tumor progression occurs, neuroendocrine tumor markers should be assessed, and repeat biopsy may be conducted to clarify the diagnosis, thereby allowing timely adjustments to the therapeutic strategy (25, 26). However, no current reports have associated TP53 and RB1 mutations with histological transformation in gastric malignancies.

In our case: (1) Preoperative enhanced abdominal CT and chest CT revealed a malignant lesion localized at the lesser curvature of the gastric fundus without additional abnormalities. (2) Multiple postoperative reviews of preoperative biopsy specimens did not identify any neuroendocrine tumor-related components. (3) After four cycles of neoadjuvant therapy, the primary tumor achieved complete pathological remission, with only two lymph nodes identified as neuroendocrine tumor metastases. (4) Regular postoperative follow-up examinations conducted over two years showed no tumor recurrence, thus ruling out occult neuroendocrine tumors. Collectively, these findings support the conclusion that gastric adenocarcinoma underwent transformation into a neuroendocrine tumor following neoadjuvant therapy. Currently, no standard treatment regimens have been reported for gastric adenocarcinoma that transforms into a neuroendocrine tumor. Drawing from treatment approaches used for transformed lung adenocarcinoma and prostate cancer, the patient underwent postoperative chemotherapy with cisplatin and etoposide (25, 27). At the most recent follow-up (18 months after surgery), the patient remained in good condition without detectable abnormalities.

## Conclusion

To date, no reported cases have documented the transformation of gastric adenocarcinoma into gastric neuroendocrine tumors following neoadjuvant chemotherapy, and the underlying mechanisms remain unclear. Further investigation involving more cases is warranted. This case report contributes to our understanding of this rare phenomenon and provides reference information for the diagnosis, treatment, and prognostic evaluation of similar cases in clinical practice.

## Data Availability

Publicly available datasets were analyzed in this study. This data can be found here: 2538395335@qq.com.
